# Iron status, development, and behavior in young children in the Pennsylvania foster care system

**DOI:** 10.1371/journal.pone.0289951

**Published:** 2023-08-17

**Authors:** Amrita Arcot, Xueyi Xing, Xiang Gao, Sarah A. Font, Laura E. Murray-Kolb

**Affiliations:** 1 Department of Nutritional Sciences, The Pennsylvania State University, University Park, PA, United States of America; 2 Evidence-to-Impact Collaborative, Social Science Research Institute, The Pennsylvania State University, University Park, PA, United States of America; 3 Department of Nutrition and Food Hygiene, School of Public Health, Fudan University, Shanghai, China; 4 Department of Sociology and Criminology, The Pennsylvania State University, University Park, PA, United States of America; 5 Department of Nutrition Science, Purdue University, West Lafayette, IN, United States of America; Emory University School of Public Health, UNITED STATES

## Abstract

**Background:**

Children in foster care are classified as a highly vulnerable population and struggle with both physical and mental health problems. Medical conditions, like poor nutritional status, remain understudied in children in foster care. To our knowledge, few studies in children in U.S. foster care have quantified the prevalence of anemia, and no studies have examined the association between anemia status and relevant developmental and behavioral outcomes.

**Objective/aims:**

(1) To determine the prevalence of anemia among children in or adopted from Pennsylvania foster care, between the ages of six months to ten years and (2) To examine if a child’s anemia status is associated with greater odds of relevant developmental and behavioral diagnoses.

**Methods:**

We conducted a secondary data analysis utilizing the *Medicaid Analytic eXtract* database between 2010–2015. Children six months–ten years were included in the analysis if they were in or had been adopted from Pennsylvania foster care. Logistic regression was used to calculate adjusted odds ratios (AOR) with 95% confidence intervals for the association between iron status and health outcomes.

**Results:**

A total of 50,311 children were included in our sample, of which 1,365 children (2.7%) were diagnosed with anemia. Children diagnosed with anemia had greater odds of delayed milestones (AOR: 2.38 [1.64–3.45]), specific delays in development (AOR: 1.59 [1.23–2.07]), adjustment disorder (AOR: 1.59 [1.06–2.39]), and irritability (AOR: 10.57 [3.36–33.25]), than children not diagnosed with anemia.

**Conclusion:**

The prevalence of anemia among children between six months–ten years in or adopted from the Pennsylvania foster care system is within the national rate of U.S. childhood anemia. Odds of several relevant developmental and behavioral diagnoses were greater among children diagnosed with anemia than children who were not.

## Introduction

Children in foster care are classified as a highly vulnerable health population, with 40–90% of children who enter foster care having a physical health problem [1–7]. The total number of children in the United States foster care system in 2020 was 407,493, and the most frequent circumstance for child removal from the home, and placement in foster care, was neglect [8]. The number of children served in Pennsylvania (PA) Foster Care remained stable from 2016 to 2019, with a moderate decrease in 2020 [9]. A total of 21,689 children were served by the PA foster care system in 2020 of which, more than half were between 0–11 years old [10]. Children who enter child welfare services frequently have undiagnosed chronic or acute medical conditions [2, 11]. The assessment of nutritional status of children in United States Foster Care is predominantly focused on macronutrient or anthropometric measures [12, 13]. Research on vitamin and nutrient status among this population is limited [6, 14–16]. This is of concern because micronutrient status impacts the development of children [17, 18]. More information is needed on the micronutrient status of children in foster care and its potential consequences.

Iron deficiency (ID) is the most common micronutrient deficiency worldwide [19]. ID, even in the absence of anemia, has implications for neurophysiological development, with downstream impacts on behavioral and cognitive outcomes [17, 18]. Animal studies support that iron is critical for proper myelination, neurogenesis, dendritogenesis, and neurochemical synthesis [20, 21]. Human studies have found that ID during infancy and early childhood could increase one’s risk of impaired cognitive, motor, behavioral, and neurological outcomes (short- and long-term) [17, 22]. As children in foster care have a high prevalence of mental and physical health abnormalities, the additional burden of poor iron status could further exacerbate suboptimal child development [1–7, 23]. Unfortunately, data on specific iron biomarkers are not readily available for this population so information on anemia must be used as a proxy, while acknowledging that multiple etiologies exist for the development of anemia.

Collectively, information on anemia status and its relationship with behavioral and developmental outcomes is lacking among children in United States Foster Care, and, as such, the following exploratory study aims to start on a state-level to determine whether further examination is warranted. The objectives are to (1) evaluate the rate of anemia among children in or adopted from the PA foster care system and (2) determine the odds of developmental and behavioral impairments among children in or adopted from the PA foster care system, with and without anemia. For our first objective, we hypothesized that the rate of anemia among our sample would be greater than the current United States rates of childhood anemia. For our second objective, we hypothesized greater odds of developmental and behavioral impairments among children with diagnosed anemia, relative to children without diagnosed anemia.

## Materials and methods

### Data source

This study is a cross-sectional analysis of anonymized Medicaid claims, utilizing the Medicaid Analytic eXtract (*MAX*) database [24]. *MAX* files contain national deidentified Medicaid enrollment and claims data from 1999–2015, with diagnoses using the International Classification of Diseases, 9^th^ and 10^th^ Revision, Clinical Modification (ICD-9-CM and ICD-10-CM, respectively). *MAX* data are made available every five years to protect patient privacy. As such, the most recently available data at the time of extraction were from 2015. Diagnostic codes were originally formatted as ICD-9-CM and were changed to ICD-10-CM in October 2015, warranting inclusion of both diagnostic terminology in the analysis. An approved study team researcher (XX) accessed *MAX* files via the Centers for Medicare and Medicaid Services’ (CMS) Virtual Research Data Center. Data were extracted for children between six months to ten years old, who were placed in PA Foster Care between January 1, 2010 to December 31, 2015. Due to restrictions in data extraction, data were limited to 2010 to observe anemia prevalence within the past ten years. Data on anemia, behavioral, and developmental impairments were all extracted using diagnostic codes from *MAX* inpatient and other therapy files. This study was declared exempt by the Institutional Review Board at The Pennsylvania State University (STUDY0001559). Informed consent by a parent and/or guardian was not needed for the use of these data.

### Criteria for selection of exposure

A child was identified with anemia based on a categorical list of diagnoses described below, and no diagnoses were identified to indicate ID without anemia. The anemia category included *chronic blood loss anemia* (ICD-9-CM: 280.0; ICD-10-CM: D50.0), *iron deficiency anemia*, *dietary* (ICD-9-CM: 280.1; ICD-10-CM: D50.9), *iron deficiency anemia*, *not elsewhere classifiable (NEC)* (ICD-9-CM: 180.8; ICD-10-CM: D50.8), *iron deficiency anemia*, *no other symptoms (NOS)* (ICD-9-CM: 280.9), *anemia*, *unspecified* or *low hematocrit* or *low hemoglobin* (ICD-9-CM: 285.9; ICD-10-CM: D64.9).

### Criteria for selection of outcomes: Behavioral

Behavioral diagnoses which either have a well-established or strongly hypothesized relationship with ID and IDA were extracted for inclusion. Diagnoses which relate to anxiety and depression [25–27] include *anxiety*, *dissociative and somatoform disorders* (ICD-9-CM: 300; ICD-10-CM: F41.9), *generalized anxiety disorder* (ICD-9-CM: 300.02; ICD-10-CM: F41.1), *depressive disorder* (ICD-9-CM: 311; ICD-10-CM: F43.2), *emotions specific to childhood or adolescence with misery and unhappiness* (ICD-9-CM: 313.1; ICD-10-CM approximate: R45.2), *prolonged depressive reaction* (ICD-9-CM: 309.1; ICD-10-CM: F43.21), and *other psychological or physical stress*, *NEC* (ICD-9-CM: V62.89; ICD-10-CM: R41.83). Other psychological or physical stress is synonymous with borderline intellectual functioning. Diagnoses which relate to impulse control and emotional regulation include [26, 28–30], *adjustment disorder* (ICD-9-CM: 309; ICD-10-CM: F43.2), *irritability* (ICD-9-CM: 799.22; ICD-10-CM: R45.4), *disruptive mood dysregulation disorder* (ICD-9-CM: 296.99; ICD-10-CM: F34.81), *impulse control disorder* (ICD-9-CM: 312.3; ICD-10-CM: F63.9), *other and unspecified special symptoms or syndromes*, *NEC* (ICD-9-CM: 307.9; ICD-10-CM: R45.1), and *other signs and symptoms involving an emotional state* (IC9-CM: 799.29; ICD-10-CM: R45.8). *Other and unspecified special symptoms or syndromes*, *NEC* is synonymous with *restlessness and agitation* and *other signs and symptoms involving an emotional state* can be compared to symptoms of nervousness.

Iron has a strongly hypothesized relationship with attention deficit disorder (ADD), attention deficit hyperactivity disorder (ADHD) [31–33], and autism spectrum disorder (ASD) [34, 35]. As such *ADHD*, *inattentive* (ICD-9-CM: 314.00; ICD-10-CM: F90.0), *ADD with hyperactivity* (ICD-9-CM: 314.00; ICD-10-CM: F90.9), and *ADHD*, *combined* (ICD-9-CM: 314.01; ICD-10-CM: F90.2) were analyzed.

### Criteria for selection of outcomes: Cognitive

The relationship between ID and IDA and impaired developmental outcomes (specifically cognition) has been well-established in the literature, with improvements in cognitive impairments when a person is provided appropriate iron fortification, depending on their age [36–40]. To capture these relationships, we included *disability*, *intellectual* (ICD-9-CM: 319; ICD-10-CM: F79), *mild intellectual disability (IQ*: *50–70)* (ICD-9-CM: 317; ICD-10-CM: F71), *moderate intellectual disability (IQ*: *35–49)* (ICD-9-CM: 318; ICD-10-CM: F71), *severe intellectual disability (IQ*: *20–34)* (ICD-9-CM: 318.1; ICD-10-CM: F72), *profound intellectual disability (IQ under 20)* (ICD-9-CM: 318.2; ICD-10-CM: F73); and *educational circumstances* (ICD-9-CM: V62.3; ICD-10-CM: Z55.9). Developmental codes included *signs and symptoms involving cognition* (ICD-9-CM: 799.5; ICD-10-CM: R41.89), *other signs and symptoms involving cognition* (ICD-9-CM: 799.59; ICD-10-CM: R41.84), and *delayed milestones* (ICD-9-CM: 783.42; ICD-10-CM: R62.0).

Three of the above diagnoses contain several sub-diagnoses (*specific delays in development* [ICD-9-CM: 315; ICD-10-CM: R625.0], *adjustment reaction* [ICD-9-CM: 309; ICD-10-CM: F43.2], and *signs and symptoms involving cognition* [ICD-9-CM: 799.5; ICD-10-CM: R418.9]). These diagnoses were treated as an overall category because their sub-diagnoses were relevant and have a strongly hypothesized relationship with poor iron status [17].

### Demographic data extraction

Demographic data were extracted for race and sex with codes from the *Centers for Medicare and Medicaid Services Chronic Conditions Data Warehouse Data Dictionary* [41, 42]. To evaluate age, children were categorized into three major groups: 6 months– 1.99 years, 2–4.99 years, and 5–10 years of age. For children in the anemia group, age was calculated as the difference between the first instance of an anemia diagnosis and the child’s birth date to indicate age at diagnosis. For children in the non-anemia group, age was calculated as the difference between the first day of the calendar year a child appeared in the *MAX* database, and the child’s birth date. A child’s age was calculated based on their first appearance in the MAX database. Due to the methodological differences in age extraction, group comparisons by age could not be conducted.

No data were available for height or weight; therefore, we extracted relevant codes for pediatric body mass index (BMI) as a proxy. Codes include *less than 5*^*th*^ *percentile for age* (ICD-9-CM: V85.51; ICD-10-CM: Z68.51); *5*^*th*^ *percentile to less than 85*^*th*^ *percentile for age* (ICD-9-CM: V85.52; ICD-10-CM: Z68.52); *85*^*th*^ *percentile to less than 95*^*th*^ *percentile for age* (ICD-9-CM: V85.53; ICD-10-CM: Z68.53), and g*reater than or equal to 95*^*th*^ *percentile for age* (ICD-9-CM: V85.54; ICD-10-CM: Z68.54).

### Data extraction, cleaning, and aggregation

Children in or adopted from PA Foster Care were captured by *MAX* uniform eligibility code “48”, which indicates Title IV-E funding. Notably, there are no codes to distinguish a child in foster care or a child recently adopted out of foster care. Title IV-E provides maintenance payments for children in foster care and adoption assistance for children who are adopted from foster care: children covered by IV-E foster care or adoption are categorically eligible for Medicaid. However, not all children in foster care or adopted from care receive IV-E funding. In 2014, Pennsylvania claimed IV-E for 44% of children in foster care and 80% of children receiving adoption subsidies [43].

Thus, the sample is comprised of children who were in Title IV-E supported foster care during the study period (2010–2015) or retained Medicaid coverage under Title IV-E after being adopted from foster care. As such, children in our sample are characterized as having experienced foster care at some point in their life. Data from the Adoption and Foster Care Analysis and Reporting System (AFCARS) were utilized to approximate the composition of our sample (adopted out of foster care versus not) [44–49]. Only children who were in PA Foster Care, with an IV-E marker were retained in the AFCARS datasets. Age in the AFCARS dataset was calculated as the difference between the child’s birth date and the first day of the fiscal year (October 1) for each year. The AFCARS dataset was used solely for the purpose of approximating our study composition (see *Discussion section*).

### Statistics

All statistical analyses were run in SAS 9.4, SAS Enterprise Guide 7.15, and RStudio, version 1.2.5033 software [50–52]. The sample of children who experienced PA Foster Care was divided into two major groups: diagnosed with anemia and not diagnosed with anemia using the ICD-9-CM and ICD-10-CM codes indicated above. Only unique instances of children with an anemia sub-diagnosis were utilized in group comparisons to prevent over-estimation of prevalence rates. For the first aim, the total number of children diagnosed with anemia were quantified and divided by the total number of children in the sample, to determine the percent prevalence of diagnosed anemia among children (6 months– 10 years) who experienced PA Foster Care, between 2010 to 2015.

For the second aim, we conducted chi-square tests to determine simple group differences. When expected sample size was less than five, a Fisher’s exact test was utilized in place of a chi-squared test. Multivariate logistic regressions were utilized to calculate adjusted odds ratios (AOR), with a 95% confidence interval, after adjusting for demographic characteristics. Covariates used in the model included sex, age, BMI, and race/ethnicity [53–57]. Secondary analysis was conducted to examine interaction effects between the above demographic characteristics and anemia, when predicting behavioral and developmental diagnoses. All statistical tests were two-sided, with an alpha level of 0.05. Statistical significance was determined by a 95% confidence interval crossing 1.00 and a *p* value < 0.05 for simple and multivariate regression. Power analysis was conducted to determine the minimum sample size required to test the study hypothesis. Results indicated that the required sample size to achieve 80% for detecting a medium effect, at a significance level of alpha = 0.05, was n = 146 for regression analysis [50]. Bonferroni correction was implemented for interaction effects, where significance was determined by a *p* value of < 0.005, to account for 10 possible interaction terms [58].

## Results

### Sample characteristics

A total of 50,311 children met the eligibility criteria for the present study. Accounting for unique instances, 1,365 children (2.71%) were diagnosed with anemia (Table 1). Diagnostic terms are not mutually exclusive, as such 1,483 total instances are reported in Table 1.

**Table 1:** Anemia diagnostic criterion and total frequency.

Diagnosis*	Diagnosed with anemia
	(total = 1,483)
Chronic blood loss anemia	8 (0.54)
Iron deficiency anemia, dietary	37 (2.49)
Iron deficiency anemia, NEC	16 (1.08)
Iron deficiency anemia, NOS	290 (19.55)
Anemia, unspecified^ƚ^	1132 (76.33)
Hematocrit, low^ƚ^	1132 (76.33)
Hemoglobin, low^ƚ^	1132 (76.33)

Values are N (%)

Abbreviations: NEC: Not elsewhere classifiable, NOS: No other symptoms

*Diagnostic terms are not mutually exclusive

^ƚ^Indicated diagnoses have the same diagnostic code and are included in the total sample once

The distribution of sex, age, race and ethnicity, and BMI are presented in Table 2. Both groups had a relatively even number of boys and girls. Children were predominantly White (anemia: 44.82%; non-anemia: 53.87%) and Black (anemia: 41.78%; non-anemia: 33.14%). Pediatric BMI data were available for 1,886 children, nearly 4% of the total sample. Of these, a majority of children were between the 5^th^ and 85^th^ percentile for age.

**Table 2:** Demographics by anemia diagnosis.

Category	Diagnosed with anemia	Not diagnosed with anemia	*P*-value^a^
**N**	1,365 (2.71)	48,946 (97.29)	
**Sex**			0.44
Male	718 (52.60)	25,214 (51.51)	
**Age**			N/A^b^
6 months to 1.99 years	411 (30.11)	12,912 (26.38)	
2 to 4.99 years	610 (44.69)	14,997 (30.64)	
5 to 10 years	344 (25.20)	21,037 (42.98)	
**Race and Ethnicity**			< 0.001
NH White	589 (44.82)	26,367 (53.87)	
NH Black	549 (41.78)	16,219 (33.14)	
Hispanic/Latino	93 (7.08)	2,973 (6.07)	
Hispanic or Latino and	26 (1.98)	1,204 (2.46)	
> 1 or more race			
Unknown	41 (3.12)	1,770 (3.62)	
Other^§^	16 (1.22)	413 (0.82)	
**BMI Classifications**			0.30^c^
Less than 5^th^	9 (10.34)	98 (5.45)	
percentile-for-age			
5^th^ percentile to less than 85^th^ percentile-for-age	49 (56.32)	1,087 (60.42)	
85^th^ percentile to less than 95^th^ percentile-for-age	12 (13.79)	262 (14.56)	
Greater than or equal to 95^th^ percentile-for-age	17 (19.54)	352 (19.57)	

Values are N (%)

^a^χ^2^ test for independence, with Yates’ correction of all factors, unless otherwise indicated

^b^Unable to determine variation because method of calculation was different between groups

^c^Fisher test for small, expected frequencies (< 5) between iron status group

^§^“Other” includes Asian or Pacific Islander, Native American or Alaskan Native, Native Hawaiian or Other Pacific Islander, and More than one race (NH or non-Latino). The above groups made up < 1% of their sub-sample

### Univariate and multivariate analysis

Of the 16 relevant behavioral diagnoses extracted, the crude odds of *adjustment disorder*, *autism spectrum disorder*, *disruptive mood dysregulation disorder*, *irritability*, *and restlessness* were 49–800% greater among children diagnosed with anemia, when compared to children not diagnosed with anemia. Of the ten developmental diagnoses extracted, the crude odds of *delayed milestones*, *intellectual disability*, *moderate*, *severe*, *and profound intellectual disability*, *and specific delays in development* were 300–630% greater among children diagnosed with anemia, when compared to children not diagnosed with anemia. Many of these crude differences lost statistical significance when adjusted for demographic variables, likely related to small case numbers for these diagnoses.

After adjusting for covariates, a child diagnosed with anemia had a nearly 60% greater odds of an *adjustment disorder* diagnosis (an overall category encompassing depressive disorder, posttraumatic stress disorder, withdrawal, and anxiety) when compared to children not diagnosed with anemia (Table 3; AOR: 1.59 [1.06–2.39]). Adjusted odds of *irritability* were ten-fold greater, among children in the anemia group, when compared with children in the not diagnosed with anemia group (AOR: 10.57 [3.36–33.25]). Adjusted odds of *delayed milestones* were approximately two-fold higher among children in the anemia group, when compared to children not diagnosed with anemia (AOR: 2.38 [1.64–3.45]). Lastly, a child diagnosed with anemia had a nearly 60% greater adjusted odds of a *specific delays in development* (overall category) when compared to children not diagnosed with anemia (AOR: 1.59 [1.23–2.07]). No other behavioral or developmental diagnosis was significant, after adjustment for demographic covariates (Table 3).

**Table 3:** Crude and adjusted odds ratio for relevant diagnoses as predicted by anemia.

Relevant behavioral diagnoses				
Diagnosis	Diagnosed with anemia, n (%), total = 1365	Not diagnosed with anemia, n (%), total = 48946	OR^†^ (95% CI)	AOR^ƚ^ (95% CI)
ADD with hyperactivity	188 (13.77)	6636 (13.56)	1.02 (0.87–1.19)	0.80 (0.43–1.46)
ADHD, inattentive	30 (2.20)	1351 (2.76)	0.79 (0.55–1.14)	1.80 (0.42–7.68)
ADHD, combined	192 (14.07)	6765 (13.82)	1.02 (0.87–1.19)	0.68 (0.36–1.29)
Adjustment disorder (overall)	258 (18.83)	627 (1.28)	**1.49 (1.30–1.71)**	**1.59 (1.06–2.39)**
Anxiety, dissociative and somatoform disorders	45 (3.30)	1277 (2.61)	1.27 (0.94–1.72)	1.38 (0.48–4.00)
Autism spectrum disorder	55 (4.03)	1264 (2.58)	**1.58 (1.20–2.09)**	1.29 (0.59–2.81)
Depressive disorder, NEC	14 (1.03)	533 (1.10)	0.94 (0.55–1.61)	1.01 (0.11–8.97)
Disruptive mood dysregulation disorder	12 (0.88)	225 (0.46)	**1.92 (1.07–3.44)**	N/A
Emotions specific to childhood or adolescence with misery and unhappiness	0 (0.00)	2 (<0.01)	N/A	N/A
Generalized anxiety disorder	8 (0.59)	274 (0.56)	1.05 (0.52–2.12)	3.52 (0.41–3.024)
Impulse control disorder	18 (1.32)	577 (1.18)	1.12 (0.70–1.80)	2.84 (0.63–12.84)
Irritability	12 (0.89)	54 (0.11)	**8.03 (4.29–15.05)**	**10.57 (3.36–33.25)**
Restlessness	10 (0.73)	145 (0.30)	**2.48 (1.31–4.73)**	2.37 (0.30–18.78)
Other psychological or physical stress, NEC	3 (0.22)	2 (<0.01)	2.56 (0.79–8.29)	2.71 (0.18–41.10)
Other signs and symptoms involving emotional state	3 (0.22)	52 (0.11)	2.07 (0.65–6.64)	1.46 (0.05–41.34)
Prolonged depressive reaction	2 (0.15)	31 (<0.01)	2.32 (0.55–9.68)	N/A
**Relevant developmental diagnoses**				
Delayed milestones	134 (9.82)	1483 (3.03)	**3.48 (2.89–4.19)**	**2.38 (1.64–3.45)**
Disability, intellectual	23 (1.68)	280 (0.57)	**2.98 (1.94–4.57)**	1.49 (0.38–5.83)
Educational circumstances	0 (0.00)	40 (<0.01)	N/A	N/A
Mild intellectual disability	7 (0.51)	194 (0.40)	1.30 (0.61–2.76)	N/A
(IQ 50–70)				
Moderate intellectual disability	7 (0.51)	78 (0.16)	**3.23 (1.49–7.01)**	N/A
(IQ 35–49)				
Other signs and symptoms involving cognition	2 (0.15)	25 (<0.01)	2.87 (0.68–12.14)	N/A
Profound intellectual disability	7 (0.51)	40 (<0.01)	**6.30 (2.82–14.09)**	3.45 (0.27–43.74)
(IQ under 20)				
Severe intellectual disability	7 (0.51)	50 (0.10)	**5.04 (2.28–11.14)**	N/A
(IQ 20–34)				
Signs and symptoms involving cognition (overall)	6 (4.40)	124 (0.25)	1.74 (0.76–3.95)	N/A
Specific delays in development (overall)	655 (47.99)	9457 (19.32)	**3.85 (3.46–4.29)**	**1.59 (1.23–2.07)**

Abbreviations: ADHD: attention deficit hyperactivity disorder, ADD: attention deficit disorder, AOR: adjusted odds ratio, IDA: iron deficiency anemia, IQ: intelligence quotient, N/A: not applicable related to a null cell value, NEC: not elsewhere classifiable, NOS: no other symptoms, OR: odds ratio, 95% CI: 95% confidence interval

^†^The reference group were children not diagnosed with anemia

^ƚ^Regressing relevant developmental and behavioral diagnoses on overall anemia, adjusted for race (ref = NH white), sex (ref = male), age (ref = 6 months– 2 years), and body mass index (ref = 5th– 85^th^ percentile)

Bold denotes statistical significance (*p* < 0.05)

*Overall* signifies an overall category with several subcategories

Secondary multivariate analysis was conducted to examine interaction effects when adjusting for sex, race/ethnicity, BMI, and age. The AOR (95% CI) estimates present in Supporting information are for behavioral and developmental diagnoses which had a significant main effect with the diagnosis of anemia.

The main effects of anemia and age were significant for 2–4 years (AOR: 0.22 [0.21–0.24]) and 5–10 years of age (AOR: 0.06 [0.05–0.06]), when predicting *specific delays in development* (S1 Table). Based on the interaction effect, those with anemia, between the ages of 2 to 4 years (AOR: 2.85 [2.17–3.71]) and 5–10 years (AOR: 2.87 [2.05–4.02]), had two-fold greater odds of *specific delays in development*, compared to children without anemia, between 6 months to 2 years of age. All other interaction effects were non-significant (*p* > 0.005).

## Discussion

To our knowledge, this is the first study which evaluates anemia among children in or adopted from the PA foster care system. Children were eligible for the study if they were in foster care (or adopted out of foster care) and were between six months to ten years of age, in 2010 to 2015. We hypothesized that the rate of anemia among children in or adopted from the PA foster care system would be greater than the current U.S. rates of childhood anemia. Of our sample, 2.7% of children were diagnosed with anemia. Analysis of NHANES data from 1988 to 1994 indicated a 3.0% prevalence of IDA among toddlers, aged one to two years [59]. A more recent NHANES analysis (2007 to 2010) [60], examined prevalence rates of anemia and IDA in children aged one to five years and found 3.2%, and 1.1% rates, respectively. Since our study sample includes children up to ten years of age, we examined an additional NHANES study, which extracted data from 2003 to 2012 [61]. The study found that 3.4% of children aged six months to four years, and 2.0% of children aged five to 11 years, had anemia, without specificity. Taken together, the U.S. prevalence of anemia among children between six months and ten years of age, is approximately 2.0–3.4%. Our sample proportion is within this approximate as opposed to being higher, which was our hypothesis. Importantly, our analysis was confined to examining anemia, as no biomarkers specific to ID were available in the ICD-9 and ICD-10 code bank. Consequently, we cannot determine the prevalence of ID alone within our sample.

Children in foster care and recently adopted children receive frequent medical visits as they are a highly vulnerable health population [62, 63]. Therefore, examination and identification of anemia (low hemoglobin) may be more likely to occur among children in foster care, relative to children in the general population. However, the prevalence of ID and IDA are likely underreported in both groups, due to lack of assessing iron-specific biomarkers (e.g., serum ferritin, transferrin saturation, soluble transferrin receptor) at the clinic visit. It is highly likely that ID among children in or adopted from foster care occurs to a greater degree than the rate of anemia. This is supported by the findings of Gupta *et al*. who examined children between one to five years of age in the U.S. and reported that the rate of ID (diagnosed by total body iron < 0 mg/kg) was two times greater than the rate of anemia (hemoglobin < 11 g/dL), and seven times greater than the rate of IDA [59]. It will be important for future studies to include iron-specific biomarkers to confirm and determine appropriate intervention strategies.

Our second hypothesis was that a higher prevalence of developmental and behavioral impairments would be present among children with diagnosed anemia, when compared to children not diagnosed with anemia. After adjustment for demographic characteristics two behavioral (*adjustment disorder and irritability*) and two developmental (*delayed milestones and specific delays in development*) diagnoses remained significant. Importantly, the sample size for some of these diagnoses were small (N < 15) so further research is warranted.

A significant interaction effect was present between anemia and age (2–4 years and 5–10 years), when predicting *specific delays in development*. This diagnostic term broadly encompasses diagnoses such as difficulties with reading and mathematics, developmental speech disorder, and delay or difficulties with coordination development [64, 65]. A portion of these diagnoses (e.g., mathematics and reading) may become apparent upon entry into school, making early detection and correction difficult. Notably, the first years of life encompass significant hippocampal and cortical development, along with myelinogenesis, dendritogenesis, and synaptogenesis [17]. ID during infancy can impact brain development with long-term effects on behavioral and developmental outcomes [17, 66]. ID can influence neurophysiological processes with the potential for irreversible impact if it is not corrected during rapid periods of growth (i.e., the perinatal period). Past studies strongly support the time-dependent role of brain iron on neurophysiological and neurochemical development [67]. One study examining iron status and cognitive performance using NHANES (1988–1994, ages six to 16) reported significantly lower math and block design scores among children with IDA, when compared to children with normal iron status [36]. The odds of scoring below average in math was two times greater in children with ID and/or anemia, compared to children with normal iron status. Such differences are illustrative of ID and its role in poor scholastic performance. Animal studies support the need for early iron repletion as a method of ameliorating long-term consequences of poor brain iron. One animal study reported improvements in dopamine activity, and epinephrine and norepinephrine concentration among rodents who had ID during gestation with early repletion [67]. The same study reported reduced dopamine activity among ID rodents without repletion, and reduced distance traveled (locomotive domain) when compared to the control.

Taken together, children with ID and IDA can be vulnerable to irreversible neurophysiological changes that will have downstream influence on behavioral, cognitive, and developmental outcomes. Greater odds of developmental delays among children two or older requires further examination into the subset of diagnostic conditions. Differences may be in part related to children entering daycare or school, where detection and reporting of developmental difficulties may be present. There is need for ongoing research in this population, with focus on the intersection of environment and health.

As mentioned above, various developmental and behavioral impairments are often diagnosed at certain ages, especially once a child begins school (e.g., autism, educational difficulties, ADHD, etc.) [53, 54]. Sex differences are also present among various neurodevelopmental disorders, including autism spectrum disorder and ADHD [55, 56]. Such diagnoses are more frequently found in males; however, this relationship remains poorly understood. Future studies should strongly consider these variables, along with relevant environmental factors, as listed above, which could influence results.

Our study has several strengths, including that it is the first study which evaluates anemia among children in or adopted from the PA foster care system. Children in foster care are a highly vulnerable population, making recruitment challenging. Utilizing a Medicaid database highlights a methodological option to understand this population, in the presence of recruitment limitations. Further, to our knowledge, this is the only study that has evaluated developmental and behavioral outcomes by anemia status, among children in and adopted from PA Foster Care. Previous literature on iron status and developmental outcomes in a population with some of the same challenges as those placed in foster care are confined to internationally adopted samples [27, 68–70], which cannot be completely generalized to children in U.S. Foster Care. The present study highlights the need for comprehensive clinical evaluations of ID and IDA, for children in U.S. Foster Care. ID, even in the absence of anemia, may have an irreversible impact on brain development [67, 71], especially when ID is present during critical periods of growth (e.g., the perinatal period). Therefore, early iron screening and prevention is preferred to attenuate risk of adverse developmental outcomes, like cognitive impairment. Lastly, our study supports further research into the feasibility of iron supplementation among children in foster care, and whether repletion could support behavioral and development outcomes. Given the population, recruitment for an intervention study would be difficult, but our findings emphasize that ongoing investigation is necessary.

Our study must be interpreted with caution due to its limitations. This study was unable to discern between ID, IDA, and anemia. Our study evaluates Medicaid claims only; therefore, we are unable to confirm if the child was correctly diagnosed with IDA and/or anemia. Additionally, the *MAX* database does not differentiate between a child presently in foster care and a child who has been adopted from the foster care system. As such, we cannot confidently report that children in our sample were in foster care during the selected study period. We can, however, speculate as to the representation of children in the foster care system versus children who have been adopted from the foster care system, in our database.

Data from AFCARS suggest that 29,720 children were likely adopted out of the Pennsylvania foster care during our study period. This roughly suggests that 60% of our sample are children who have been adopted out of foster care, and the remaining 40% were in foster care, during our study period. The length of time a child was in foster care, prior to our data extraction, is also unknown. Consequently, the clinical picture of a child who has recently entered foster care may not be captured in our analysis. Due to resource limitations, we were unable to determine the timings of diagnosis. Understanding the sequence of events (such as the diagnosis of anemia relative to the diagnosis of irritability or ADHD, for example) would provide a greater understanding of the association between anemia and these relevant behavioral and developmental diagnoses and is crucial for future studies. Finally, as mentioned above, we were unable to determine ID and we suspect that the rate of IDA may not have been adequately captured in our analysis. Since the rate of ID is likely higher than the rate of anemia, we would suspect a more robust sample if these diagnoses were specifically identified. It is also important to note that other drivers of behavioral and developmental issues, such as an unstable home, reason for child displacement, and number of foster care entries may be confounding the results. Abuse type, like neglect or emotional abuse, can negatively impact cognitive outcomes like visuospatial processing and memory [72]. As such, various environmental measures require further examination to determine if they mediate the association between anemia and behavioral and developmental outcomes seen here.

Environmental factors are known to impact a child’s behavioral and developmental outcomes but our findings suggest that nutrient deficiencies, such as iron deficiency, likely contribute as well. In addition to a nutrient deficiency contributing directly to poor behavioral and developmental outcomes, the deficiency may also contribute indirectly by influencing a child’s resiliency in stressful environmental circumstances. Therefore, an intervention that provides iron supplements to such a child may support a better response to other intervention strategies (such as promoting a child’s interaction with their environment). Cumulatively, children in the welfare system face a multitude of environmental, social, and health-related obstacles. Alleviating at least one burden from this highly vulnerable pediatric population with a simple and economical solution–such as iron supplementation–warrants ongoing investigation.

## Conclusion

The present study identified the prevalence of anemia among children in or adopted from PA Foster Care, a rate that was not previously known, and was found to be similar to the prevalence of anemia in the general US population. Importantly, in this sample, children with anemia had higher odds of delayed milestones, specific delays in development, adjustment disorder, and irritability, than children not diagnosed with anemia.

Children in foster care are highly vulnerable to both physical and mental health issues [1–7]. As such, identification of clinical needs with timely intervention is imperative. This includes the proper diagnosis and treatment of iron deficiency, which has a simple and affordable treatment (iron supplementation) that can potentially improve developmental and behavioral issues. Our study supports the need for further investigation into iron treatment, and how it may impact behavioral and development outcomes in this medically vulnerable pediatric population. Future studies on ID and IDA, among children in U.S. Foster Care, are needed to better understand risks of this population. If utilizing a Medicaid data for a future cross-sectional study, extracting diagnoses as an overall category may be one way to provide a robust sample size for analysis. Further, obtaining the sequence of diagnostic events will be critical in understanding the relationship between ID and/or IDA and relevant behavioral and developmental outcomes. Ideally, human-subjects research will be conducted, with the recruitment of children presently in foster care. A prospective cohort study of children recently entering foster care, with the collection of specific iron biomarkers and behavioral and/or developmental outcomes would inform the need for early ID screening and timely interventions, to potentially dismantle one burden faced by this vulnerable pediatric population.

## Supporting information

S1 Table AORs and moderating effects for diagnoses with a significant main effect between relevant behavioral/developmental diagnoses and anemia. (DOCX)

## References

[1] Flaherty EG, Weiss H. Medical evaluation of abused and neglected children. Arch Pediatr Adolesc Med. 1990;144(3):330–4. doi: 10.1001/archpedi.1990.02150270080030 2305740

[2] Chernoff R, Combs-Orme T, Risley-Curtiss C, Heisler A. Assessing the health status of children entering foster care. Pediatrics. 1994;93(4):594–601. 8134214

[3] Hochstadt NJ, Jaudes PK, Zimo DA, Schachter J. The medical and psychosocial needs of children entering foster care. Child Abuse and Neglect. 1987;11(1):53–62. doi: 10.1016/0145-2134(87)90033-0 3828875

[4] Fussell JJ, Evans LD. Medical status of school-age children reentering foster care. Child Maltreat. 2009;14(4):382–6. doi: 10.1177/1077559508326222 19047477

[5] Takayama JI, Wolfe E, Coulter KP. Relationship between reason for placement and medical findings among children in foster care. Pediatrics. 1998;101(2):201–7. doi: 10.1542/peds.101.2.201 9445492

[6] Leslie LK, Gordon JN, Meneken L, Premji K, Michelmore KL, Ganger W. The physical, developmental, and mental health needs of young children in child welfare by initial placement type. J Dev Behav Pediatr. 2005;26(3):177–85. doi: 10.1097/00004703-200506000-00003 15956866 PMC1550710

[7] Horwitz SM, Owens P, Simms MD. Specialized assessments for children in foster care. pediatrics. Pediatrics. 2000;106(1):59–66. 10878150 10.1542/peds.106.1.59

[8] U.S. Department of Health and Human Services, Administration for Children and Families, Administration on Children, Youth and Families. Adoption and Foster Care Analysis and Reporting System (AFCARS) FY 2020 data [Internet]. Children’s Bureau; 2021. Report No. 28. Available from: https://www.acf.hhs.gov/cb/report/afcars-report-28

[9] Pennsylvania Partnerships for Children. State of child welfare 2019—Pennsylvania [Internet]. Pennsylvania Partnerships for Children; 2019. Available from: papartnerships.org.

[10] Pennsylvania Partnerships for Children. State of child welfare 2021—Pennsylvania. 2021:1–10.

[11] Simms MD, Dubowitz H, Szilagyi MA. Health care needs of children in the foster care system. Pediatrics. 2000;106(4):909–18. 11044143

[12] Regber S, Jormfeldt H. Foster homes for neglected children with severe obesity—debated but rarely studied. Acta Paediatr. 2019;108(11):1955–64. doi: 10.1111/apa.14902 31199006

[13] Tooley UA, Makhoul Z, Fisher PA. Nutritional status of foster children in the U.S.: Implications for cognitive and behavioral development. Child Youth Serv Rev. 2016;70:369–74. doi: 10.1016/j.childyouth.2016.10.027 28626279 PMC5472390

[14] Greiner MV, Beal SJ, Nause K, Staat MA, Dexheimer JW, Scribano PV. Laboratory screening for children entering foster care. Pediatrics. 2017;140(6):e20163778. doi: 10.1542/peds.2016-3778 29141915

[15] DuRousseau PC, Moquette-Magee E, Disbrow D. Children in foster care: are they at nutritional risk? J Am Diet Assoc. 1991;91(1):1–3. 1869764

[16] Wojcicki AV, George PE, Palzer EF, Brearley AM, Gustafson KL, Eckerle JK. Vitamin D deficiency in a Minnesota-based foster care population: A cross sectional study. Child Youth Serv Rev. 2020;119:105611. doi: 10.1016/j.childyouth.2020.105611 33162630 PMC7643840

[17] Lozoff B, Beard J, Connor J, Felt B, Georgieff M. Long-lasting neural and behavioral effects of iron deficiency in infancy. Nutr Rev. 2006;64(5):S34–43. doi: 10.1301/nr.2006.may.s34-s43 16770951 PMC1540447

[18] Lozoff B. Iron deficiency and child development. Food Nutr Bull. 2007;28(4):S560–71. doi: 10.1177/15648265070284S409 18297894

[19] de Benoist B, World Health Organization, Centers for Disease Control and Prevention (U.S.). Worldwide prevalence of anaemia 1993–2005 of: WHO global database of anaemia. World Health Organization; 2008. Available from: http://whqlibdoc.who.int/publications/2008/9789241596657_eng.pdf

[20] Beard JL, Connor JR. Iron status and neural functioning. Annu Rev Nutr. 2003;23:41–58. doi: 10.1146/annurev.nutr.23.020102.075739 12704220

[21] Wade QW, Chiou B, Connor JR. Iron uptake at the blood-brain barrier is influenced by sex and genotype. Adv Pharmacol. 2019;84:123–45. doi: 10.1016/bs.apha.2019.02.005 31229168

[22] Pivina L, Semenova Y, Doşa MD, Dauletyarova M, Bjørklund G. Iron deficiency, cognitive functions, and neurobehavioral disorders in children. J Mol Neurosci. 2019;68(1):1–10. doi: 10.1007/s12031-019-01276-1 30778834

[23] Harman JS, Childs GE, Kelleher KJ. Mental health care utilization and expenditures by children in foster care. Arch Pediatr Adolesc Med. 2000;154:1114–7. doi: 10.1001/archpedi.154.11.1114 11074852

[24] Williams S, Baugh D. Medicaid Analytic eXtract Data (MAX): Providing data to researchers and policy analysts. centers for Medicare & Medicaid services; 2016. Available from: https://www.cms.gov/Research-Statistics-Data-and-Systems/Computer-Data-and-Systems/MedicaidDataSourcesGenInfo/MAXGeneralInformation

[25] East P, Lozoff B, Blanco E, Delker E, Delva J, Encina P, et al. Infant iron deficiency, child affect, and maternal unresponsiveness: testing the long-term effects of functional isolation. Dev Psychol. 2017;53(12):2233–44. doi: 10.1037/dev0000385 28933876 PMC5705270

[26] Corapci F, Calatroni A, Kaciroti N, Jimenez E, Lozoff B. Longitudinal evaluation of externalizing and internalizing behavior problems following iron deficiency in infancy. J Pediatr Psychol. 2010;35(3):296–305. doi: 10.1093/jpepsy/jsp065 19736288 PMC2842097

[27] Doom JR, Richards B, Caballero G, Delva J, Gahagan S, Lozoff B. Infant iron deficiency and iron supplementation predict adolescent internalizing, externalizing, and social problems. J Pediatr. 2018;195:199–205. doi: 10.1016/j.jpeds.2017.12.008 29395182 PMC5869133

[28] East P, Delker E, Lozoff B, Delva J, Castillo M, Gahagan S. Associations Among infant iron deficiency, childhood emotion and attention regulation, and adolescent problem behaviors. Child Dev. 2018;89(2):593–608. doi: 10.1111/cdev.12765 28233303 PMC5569004

[29] Chang S, Wang L, Wang Y, Brouwer ID, Kok FJ, Lozoff B, et al. Iron-deficiency anemia in infancy and social emotional development in preschool-aged Chinese children. Pediatrics. 2011;127(4):e927–33. doi: 10.1542/peds.2010-1659 21402624

[30] Wachs TD, Pollitt E, Cueto S, Jacoby E, Creed-Kanashiro H. Relation of neonatal iron status to individual variability in neonatal temperament. Dev Psychobiol. 2005;46(2):141–53. doi: 10.1002/dev.20049 15732057

[31] Konofal E, Cortese S. Lead and neuroprotection by iron in ADHD. Environ Health Perspect. 2007;115(8):398–9. doi: 10.1289/ehp.10304 17687422 PMC1940080

[32] Konofal E, Lecendreux M, Arnulf I, Mouren MC. Iron deficiency in children with attention-deficit/hyperactivity disorder. Arch Pediatr Adolesc Med. 2004;158:1113–5. doi: 10.1001/archpedi.158.12.1113 15583094

[33] Juneja M, Jain R, Singh V, Mallika V. Iron deficiency in Indian children with attention deficit hyperactivity disorder. Indian Pediatr. 2010 Nov;47(11):955–8. doi: 10.1007/s13312-010-0160-9 20453262

[34] Latif A, Heinz P, Cook R. Iron deficiency in autism and Asperger syndrome. Autism. 2002;6(1):103–14. doi: 10.1177/1362361302006001008 11918106

[35] Dosman CF, Drmic IE, Brian JA, Senthilselvan A, Harford M, Smith R, et al. Ferritin as an indicator of suspected iron deficiency in children with autism spectrum disorder: prevalence of low serum ferritin concentration. Dev Med & Child Neurol. 2007;48(12):1008–9. 10.1017/S0012162206232225 17109795

[36] Halterman JS, Kaczorowski JM, Aligne CA, Auinger P, Szilagyi PG. Iron deficiency and cognitive achievement among school-aged children and adolescents in the United States. Pediatrics. 2001;107(6):1381–6. doi: 10.1542/peds.107.6.1381 11389261

[37] Pollitt E. Iron deficiency and cognitive function. Annu Rev Nutr. 1993;13(1):521–37. doi: 10.1146/annurev.nu.13.070193.002513 8369157

[38] Lozoff B, Castillo M, Clark MK, Smith BJ. Iron-fortified vs low-iron infant formula: developmental outcome at 10 years. Arch Pediatr Adolesc Med. 2012;166(3):208. doi: 10.1001/archpediatrics.2011.197 22064877 PMC3312311

[39] Gahagan S, Delker E, Blanco E, Burrows R, Lozoff B. Randomized controlled trial of iron-fortified versus low-iron infant formula: developmental outcomes at 16 years. J Pediatr. 2019;212:124–30. 31253407 10.1016/j.jpeds.2019.05.030 PMC7152502

[40] Scott SP, Murray-Kolb LE, Wenger MJ, Udipi SA, Ghugre PS, Boy E, et al. Cognitive performance in Indian school-going adolescents is positively affected by consumption of iron-biofortified pearl millet: a 6-month randomized controlled efficacy trial. J Nutr. 2018;148(9):1462–71. doi: 10.1093/jn/nxy113 30016516

[41] Centers for Medicare & Medicaid Services. CMS Chronic Conditions Data Warehouse (CCW) data dictionary: Medicaid Analytic eXtract (MAX) Personal Summary (PS) record. Chronic Conditions Data Warehouse; 2019. Available from: https://www2.ccwdata.org/web/guest/data-dictionaries

[42] Research Data Assistance Center, Center for Medicare and Medicaid Services, University of Minnesota School of Public Health, Health Policy, and Management. Race/ethnicity (from MSIS) [Internet]. Research Data Assistance Center. 2021. Available from: https://resdac.org/cms-data/variables/raceethnicity-msis

[43] Trends Child. Child Welfare Financing SFY 2014: Pennsylvania [Internet]. Child Trends; 2014. Available from: https://www.childtrends.org/wp-content/uploads/2016/10/Child-Welfare-Financing-SFY2014_Pennsylvania.pdf

[44] Children’s Bureau, Administration on Children, Youth and Families, Administration for Children and Families, U. S. Department of Health and Human Services. Adoption and Foster Care Analysis and Reporting System (AFCARS), Foster Care File 2010 [Dataset] [Internet]. National Data Archive on Child Abuse and Neglect; Report No.: 163. Available from: 10.34681/ZVQ8-NE14

[45] Children’s Bureau, Administration on Children, Youth and Families, Administration for Children and Families, U. S. Department of Health and Human Services. Adoption and Foster Care Analysis and Reporting System (AFCARS), Foster Care File 2011 [Dataset] [Internet]. National Data Archive on Child Abuse and Neglect; Report No.: 167. Available from: 10.34681/CE1A-YJ74

[46] Children’s Bureau, Administration on Children, Youth and Families, Administration for Children and Families, U. S. Department of Health and Human Services. Adoption and Foster Care Analysis and Reporting System (AFCARS), Foster Care File 2012 [Dataset] [Internet]. National Data Archive on Child Abuse and Neglect; Report No.: 176. Available from: 10.34681/4NEW-AH94

[47] Children’s Bureau, Administration on Children, Youth and Families, Administration for Children and Families, U. S. Department of Health and Human Services. Adoption and Foster Care Analysis and Reporting System (AFCARS), Foster Care File 2013 [Dataset] [Internet]. National Data Archive on Child Abuse and Neglect; Report No.: 187. Available from: 10.34681/4RN6-HT82

[48] Children’s Bureau, Administration on Children, Youth and Families, Administration for Children and Families, U. S. Department of Health and Human Services. Adoption and Foster Care Analysis and Reporting System (AFCARS), Foster Care File 2014 [Dataset] [Internet]. National Data Archive on Child Abuse and Neglect; Report No.: 192. Available from: 10.34681/E3CC-JC75

[49] Children’s Bureau, Administration on Children, Youth and Families, Administration for Children and Families, U. S. Department of Health and Human Services. Adoption and Foster Care Analysis and Reporting System (AFCARS), Foster Care File 2015 [Dataset] [Internet]. National Data Archive on Child Abuse and Neglect; Report No.: 200. Available from: 10.34681/XBTD-2H02

[50] SAS [Computer Software]. Version 9.4. Cary, NC. SAS Institute.

[51] R Core Team. R: A language and environment for statistical computing. R Foundation for Statistical Computing [Internet]. Vienna, Austria; 2020. Available from: https://www.R-project.org/

[52] Aragon TJ, Omidpanah A. Package “epitools” [Internet]. CRAN; 2020. Available from: https://cran.r-project.org/web/packages/epitools/index.html

[53] Baird G, Cass H. Diagnosis of autism. Brit Med J. 2003;327(7413):488–93. doi: 10.1136/bmj.327.7413.488 12946972 PMC188387

[54] Clinic Mayo. Attention-deficit/hyperactivity disorder (ADHD) in children. Mayo Clinic. Available from: https://www.mayoclinic.org/diseases-conditions/adhd/diagnosis-treatment/drc-20350895?p=1

[55] Werling DM, Geschwind DH. Sex differences in autism spectrum disorders: Curr Opin Neurol. 2013;26(2):146–53 10.1097/WCO.0b013e32835ee548 PMC4164392 23406909

[56] May T, Adesina I, McGillivray J, Rinehart NJ. Sex differences in neurodevelopmental disorders. Curr Opin Neurol. 2019;32(4):622–6. doi: 10.1097/WCO.0000000000000714 31135460

[57] McClung JP, Karl JP. Iron deficiency and obesity: the contribution of inflammation and diminished iron absorption. Nutr Rev. 2009;67(2):100–4. doi: 10.1111/j.1753-4887.2008.00145.x 19178651

[58] Armstrong RA. When to use the Bonferroni correction. Ophthalmic and Physiological Optics. 2014;34(5):502–8. doi: 10.1111/opo.12131 24697967

[59] Looker AC, Carroll D, Gunter EW, Johnson CL. Prevalence of iron deficiency in the United States. J Am Med Assoc. 1997;277(12):973–6. doi: 10.1001/jama.1997.03540360041028 9091669

[60] Gupta P, Perrine C, Mei Z, Scanlon K. Iron, anemia, and iron deficiency anemia among young children in the United States. Nutrients. 2016;8(6):330. doi: 10.3390/nu8060330 27249004 PMC4924171

[61] Le CHH. The prevalence of anemia and moderate-severe anemia in the US population (NHANES 2003–2012). PLoS ONE. 2016;11(11):e0166635. doi: 10.1371/journal.pone.0166635 27846276 PMC5112924

[62] Szilagyi MA, Council on Foster Care, Adoption, and Kinship Care, Committee on Adolescence, and Council on Early Childhood. Health care issues for children and adolescents in foster care and kinship care. Pediatrics. 2015;136(4):e1131–40. 26416941 10.1542/peds.2015-2655

[63] Jones VF, Schulte EE, Council on Foster Care, Adoption, and Kinship Care. Comprehensive health evaluation of the newly adopted child. Pediatrics. 2019;143(5):e20190657. 31036671 10.1542/peds.2019-0657

[64] Alkaline Software. About ICD9Data.com and ICD10Data.com [Internet]. ICD10Data.com. Available from: http://www.icd10data.com/

[65] Alkaline Software. About ICD9Data.com and ICD10Data.com [Internet]. ICD9Data.com. Available from: http://www.icd9data.com/

[66] Jorgenson LA, Sun M, O’Connor M, Georgieff MK. Fetal iron deficiency disrupts the maturation of synaptic function and efficacy in area CA1 of the developing rat hippocampus. Hippocampus. 2005;15(8):1094–102. doi: 10.1002/hipo.20128 16187331

[67] Unger EL, Paul T, Murray-Kolb LE, Felt B, Jones BC, Beard JL. Early iron deficiency alters sensorimotor development and brain monoamines in rats. J Nutr. 2007;137(1):118–24. doi: 10.1093/jn/137.1.118 17182811

[68] Fuglestad AJ, Kroupina MG, Johnson DE, Georgieff MK. Micronutrient status and neurodevelopment in internationally adopted children. Acta Paediatr. 2016;105(2):e67–76. doi: 10.1111/apa.13234 26439893

[69] Fuglestad AJ, Lehmann AE, Kroupina MG, Petryk A, Miller BS, Iverson SL, et al. Iron deficiency in international adoptees from Eastern Europe. J Pediatr. 2008;153(2):272–7. doi: 10.1016/j.jpeds.2008.02.048 18534235

[70] Fuglestad AJ, Georgieff MK, Iverson SL, Miller BS, Petryk A, Johnson DE, et al. Iron deficiency after arrival is associated with general cognitive and behavioral impairment in post-institutionalized children adopted from Eastern Europe. Matern Child Health J. 2013;17(6):1080–7. doi: 10.1007/s10995-012-1090-z 22872286

[71] Beard J, Erikson KM, Jones BC. Neonatal iron deficiency results in irreversible changes in dopamine function in rats. J Nutr. 2003;133(4):1174–9. doi: 10.1093/jn/133.4.1174 12672939

[72] Pears K, Fisher PA. Developmental, cognitive, and neuropsychological functioning in preschool-aged foster children: associations with prior maltreatment and placement history. J Dev Behav Pediatr. 2005;26(2):1–11. doi: 10.1097/00004703-200504000-00006 15827462

