# Supplementary material for: Iron status, development, and behavior in young children in the Pennsylvania foster care system
Source: PLoS One. 2023 Aug 17;18(8):e0289951. doi: 10.1371/journal.pone.0289951 (PMC10434919; doi:10.1371/journal.pone.0289951)
Supplement: S1 Table — (DOCX) [file pone.0289951.s001.docx]

# Supporting information

| **S1 Table. AORs and moderating effects for diagnoses with a significant main effect between relevant behavioral/developmental diagnoses and anemia** | | | | |
| --- | --- | --- | --- | --- |
| **Diagnoses Based On:** | | **Estimate (SE)** | **AOR (95% CI)** | ***p-*value** |
| **Adjustment disorder** | | | | |
| Anemia | | 0.46 (0.21) | 1.59 (1.06 – 2.39) | 0.03 |
| Female | | -0.02 (0.03) | 0.98 (0.93 – 1.03) | 0.47 |
| Race | | | | |
| NH Black | | -0.18 (0.03) | 0.83 (0.79 – 0.88) | < 0.001 |
| Hispanic | | -0.02 (0.05) | 0.98 (0.89 – 1.08) | 0.64 |
| Other^§^ | | 0.06 (0.14) | 1.06 (0.80 – 1.08) | 0.69 |
| Unknown | | -0.25 (0.08) | 0.78 (0.67 – 0.91) | 0.002 |
| Age | | | | |
| 2 – 4 years | | 1.14 (0.04) | 3.13 (2.87 – 3.41) | < 0.001 |
| 5 – 10 years | | 1.17 (0.04) | 3.21 (2.95 – 3.48) | < 0.001 |
|  | | BMI |  |  |
| 0 – 5^th^ percentile | | 0.90 (0.23) | 2.45 (1.55 – 3.89) | < 0.001 |
| 85 – 95^th^ percentile | | 0.56 (0.17) | 1.76 (1.27 – 2.43) | 0.002 |
| Greater than 95^th^ percentile | | 0.66 (0.13) | 1.93 (1.49 – 2.49) | < 0.001 |
| IDA*Race | | | | |
| NH Black | -0.06 (0.16) | | 0.95 (0.69 – 1.30) | 0.73 |
| Unknown | -0.23 (0.47) | | 0.79 (0.31 – 2.00) | 0.62 |
| IDA*Age | | | | |
| 2 – 4 years | | -0.26 (0.21) | 0.77 (0.51 – 1.18) | 0.23 |
| 5 – 10 years | | 0.40 (0.22) | 1.49 (0.97 – 2.29) | 0.07 |
| IDA*BMI | | | | |
| 0 – 5^th^ percentile | | -0.78 (0.86) | 0.46 (0.09 – 2.48) | 0.37 |
| 85 – 95^th^ percentile | | 1.95 (0.83) | 7.02 (1.38 – 35.77) | 0.02 |
| Greater than 95^th^ percentile | | -0.35 (0.63) | 0.70 (0.21 – 2.39) | 0.57 |
| **Irritability** | | | | |
| IDA | | 2.36 (0.58) | 10.57 (3.36 – 33.25) | < 0.001 |
| Female | | -0.39 (0.28) | 0.68 (0.39 – 1.18) | 0.17 |
| Race | | | | |
| NH Black | | -0.38 (0.32) | 0.69 (0.37 – 1.28) | 0.24 |
| Hispanic | | -0.07 (0.48) | 0.93 (0.36 – 2.38) | 0.88 |
| Other^§^ | | -12.07 (579.50) | 0.00 (0.00 – INF) | 0.98 |
| Unknown | | -0.85 (1.02) | 0.43 (0.06 – 3.14) | 0.40 |
| Age | | | | |
| 2 – 4 years | | -0.62 (0.33) | 0.54 (0.28 – 1.03) | 0.06 |
| 5 – 10 years | | -0.94 (0.33) | 0.39 (0.20 – 0.75) | < 0.01 |
| BMI | | | | |
| 0 – 5^th^ percentile | | 2.09 (1.02) | 8.11 (1.10 – 59.52) | 0.04 |
| 85 – 95^th^ percentile | | -12.54 (1090.00) | 0.00 (0.00 – INF) | 0.99 |
| Greater than 95^th^ percentile | | -12.46 (888.60) | 0.00 (0.00 – INF) | 0.99 |
| IDA*Age | | | | |
| 5 – 10 years | | -0.19 (0.88) | 0.83 (0.15 – 4.62) | 0.83 |
| IDA*BMI | | | | |
| 0 – 5^th^ percentile | | -12.82 (1272.40) | 0.00 (0.00 – INF) | 0.99 |
| **Delayed milestones** | | | | |
| IDA | | 0.87 (0.19) | 2.38 (1.64 – 3.45) | < 0.001 |
| Female | | -0.25 (0.05) | 0.78 (0.70 – 0.86) | < 0.001 |
| Race | | | | |
| NH Black | | -0.18 (0.06) | 0.84 (0.74 – 0.94) | < 0.01 |
| Hispanic | | -0.04 (0.10) | 0.96 (0.79 – 1.16) | 0.67 |
| Other^§^ | | 0.09 (0.28) | 1.09 (0.63 – 1.89) | 0.76 |
| Unknown | | 0.17 (0.13) | 1.18 (0.92 – 1.52) | 0.20 |
| Age | | | | |
| 2 – 4 years | | -1.53 (0.07) | 0.22 (0.19 – 0.25) | < 0.001 |
| 5 – 10 years | | -3.07 (0.11) | 0.05 (0.04 – 0.06) | < 0.001 |
| BMI | | | | |
| 0 – 5^th^ percentile | | 0.90 (0.38) | 2.45 (1.15 – 5.19) | 0.02 |
| 85 – 95^th^ percentile | | -0.67 (0.59) | 0.51 (0.16 – 1.63) | 0.26 |
| Greater than 95^th^ percentile | | -0.24 (0.42) | 0.78 (0.34 – 1.78) | 0.56 |
| IDA*Sex | | | | |
| Female | | -0.12 (0.20) | 0.89 (0.60 – 1.32) | 0.56 |
| IDA*Race | | | | |
| NH Black | | 0.33 (0.22) | 1.40 (0.90 – 2.16) | 0.13 |
| IDA*Age | | | | |
| 2 – 4 years | | 0.44 (0.21) | 1.56 (1.03 – 2.36) | 0.04 |
| 5 – 10 years | | 0.4 (0.45) | 1.5 (0.65 – 3.73) | 0.32 |
| IDA*BMI | | | | |
| 0 – 5^th^ percentile | | -11.29 (182.9) | N/A | 0.95 |
| **Specific delays in development** | | | | |
| IDA | | 0.47 (0.13) | 1.59 (1.23 – 2.07) | < 0.001 |
| Female | | -0.37 (0.03) | 0.69 (0.65 – 0.72) | < 0.001 |
| Race | | | | |
| NH Black | | -0.16 (0.03) | 0.85 (0.81 – 0.90) | < 0.001 |
| Hispanic | | -0.15 (0.05) | 0.86 (0.78 – 0.94) | < 0.01 |
| Other^§^ | | -0.11 (0.14) | 0.90 (0.68 – 1.16) | 0.44 |
| Unknown | | 0.02 (0.07) | 1.02 (0.89 – 1.16) | 0.79 |
| Age | | | | |
| 2 – 4 years | | -1.50 (0.03) | 0.22 (0.21 – 0.24) | < 0.001 |
| 5 – 10 years | | -2.84 (0.04) | 0.06 (0.05 – 0.06) | < 0.001 |
| BMI | | | | |
| 0 – 5^th^ percentile | | 0.69 (0.24) | 2.00 (1.25 – 3.19) | < 0.01 |
| 85 – 95^th^ percentile | | 0.26 (0.19) | 1.30 (0.90 – 1.87) | 0.16 |
| Greater than 95^th^ percentile | | 0.31 (0.15) | 1.36 (1.01 – 1.84) | 0.04 |
| IDA*Sex | | | | |
| Female | | 0.20 (0.12) | 1.22 (0.97 – 1.53) | 0.09 |
| IDA*Race | | | | |
| NH Black | | 0.33 (0.13) | 1.39 (1.08 – 1.79) | 0.01 |
| Hispanic | | 0.13 (0.22) | 1.14 (0.74 – 1.74) | 0.56 |
| IDA*Age | | | | |
| 2 – 4 years | | 1.04 (0.14) | 2.84 (2.17 – 3.71) | < 0.001 |
| 5 – 10 years | | 1.05 (0.17) | 2.87 (2.05 – 4.02) | < 0.001 |
| IDA*BMI | | | | |
| 0 – 5^th^ percentile | | -1.31 (0.77) | 0.27 (0.06 – 1.22) | 0.09 |
| Greater than 95^th^ percentile | | -0.20 (0.60) | 0.82 (0.25 – 2.66) | 0.74 |
| Model adjusted for race (ref = NH white), sex (ref = male), age (ref = 6 months – 2 years), and body mass index (ref = 5^th^ – 85^th^ percentile)  Abbreviations: AOR: adjusted odds ratio, BMI: body mass index, IDA: iron deficiency anemia, INF: infinity, N/A: not applicable as related to a null cell value, NH: non-Hispanic, Ref: reference, SE: standard error, 95% CI: 95% confidence interval  ^§^“Other” includes Asian or Pacific Islander, Native American or Alaskan Native, Native Hawaiian or Other Pacific Islander, and More than one race (NH or non-Latino). The above groups made up < 1% of their sub-sample  Main effects are significant if *p* < 0.05  Interaction effects are significant if *p* < 0.005 | | | | |
